# Perioperative Management of a Patient With Severe Bullous Emphysema

**DOI:** 10.7759/cureus.89700

**Published:** 2025-08-09

**Authors:** Ruiyang Huang, Jacqueline Ragheb

**Affiliations:** 1 Department of Anesthesia, University of Miami Miller School of Medicine, Miami, USA; 2 Department of Anesthesia, Perioperative Medicine, and Pain Management, University of Miami, Miami, USA

**Keywords:** anesthesia for high-risk patients, bullous emphysema, chronic obstructive pulmonary disease (copd), emphysematous bullae, giant bulla, perioperative medicine, regional anesthesia, type of anesthesia

## Abstract

Patients with severe emphysematous bullae present significant anesthetic challenges, especially during laparoscopic or robotic-assisted surgeries, where positive pressure ventilation and pneumoperitoneum increase the risk of barotrauma. Rupture of a bulla can cause life-threatening tension pneumothorax, and intervention may be delayed in robotic cases due to limited access.

A 62-year-old male patient with chronic obstructive pulmonary disease (COPD) and extensive bilateral bullous disease was scheduled for robotic-assisted laparoscopic inguinal hernia repair. Preoperative imaging revealed near-complete replacement of the upper right lung by giant bullae and substantial left-sided involvement. After a multidisciplinary discussion, the robotic approach was deemed unsafe due to the risk of bulla rupture during insufflation and positive pressure ventilation. Surgery was converted to an open repair under spinal anesthesia, with monitored anesthesia care using a propofol infusion. The patient remained hemodynamically stable, required no intraoperative airway intervention, and recovered without complication.

This case highlights critical perioperative considerations in patients with bullous disease, including the limitations of chest tubes in decompressing a ruptured bulla with large bronchial communication, and the importance of individualized risk assessment beyond standard scoring systems. Preoperative imaging, multidisciplinary planning, and consideration of regional anesthesia can mitigate catastrophic complications. While alternative approaches such as regional blocks may offer a safer alternative for these patients, lung-protective ventilation strategies and avoidance of nitrous oxide are well-described for cases requiring general anesthesia.

Patients with severe bullous emphysema require tailored perioperative planning. Early recognition, surgical approach modification, and the use of regional anesthesia can prevent life-threatening complications in high-risk non-thoracic surgeries.

## Introduction

Patients with severe bullous emphysema present a unique anesthetic challenge, particularly when general anesthesia and positive pressure ventilation are required. Bullae, which are defined as air-filled spaces larger than 1 cm within the lung parenchyma, are compliant and prone to barotrauma and rupture under positive pressure [1,2]. This risk is significantly amplified during procedures involving carbon dioxide (CO_2_) pneumoperitoneum insufflation due to elevated intrathoracic pressures and ventilatory demands due to CO_2_ absorption [3,4]. Rupture of an emphysematous bullae can be life-threatening and cause tension pneumothorax and cardiovascular collapse if not quickly recognized and treated [5,6]. 

While literature exists on managing patients with bullous disease undergoing surgery, fewer reports describe the perioperative approach to patients with bullous emphysema undergoing non-thoracic, laparoscopic procedures. Even fewer address decision-making in the context of robotic-assisted surgeries, where access to the patient’s thorax during critical intraoperative events may be significantly delayed due to the physical constraints of the robotic system [6]. 

In this report, we present the case of a patient with a planned robotic-assisted right inguinal hernia repair who had extensive emphysematous bullae due to chronic obstructive pulmonary disease (COPD), which was preemptively converted to an open procedure under spinal anesthesia following a risk assessment. This case highlights the importance of recognizing the mechanical and physiological limitations imposed by the robotic surgical modality and the patient's anatomy. Additionally, it discusses the unique perioperative management considerations for patients with this condition, including preoperative risk assessment strategies, multidisciplinary discussions, modifications of the surgical approach, and intraoperative risk mitigation strategies such as the choice of anesthetic techniques and the use of lung-protective strategies if general anesthesia is necessary.

## Case presentation

Patient history 

A 62-year-old male patient with a history of COPD, bipolar disorder, hypertension, and schizophrenia presented to the emergency department with recurrent right inguinal pain over the past nine months. He had been managing with over-the-counter acetaminophen and wanted a general surgery referral for surgery. He was referred to the acute care surgery service for further evaluation. 

Preoperative evaluation 

In the surgical clinic, he was found to have a reducible right inguinal hernia and a small reducible umbilical hernia measuring less than 3 cm. Robotic-assisted laparoscopic right inguinal hernia repair with mesh and laparoscopic repair of the umbilical hernia were recommended to him. There were no recent COPD exacerbations or baseline oxygen requirements. 

Respiratory History

The patient had a documented history of COPD and severe emphysematous bullae. His social history was significant for a 40-pack year tobacco history, as well as marijuana and crack cocaine use. He was first referred to pulmonology four years ago after presenting to the emergency department with right shoulder pain from a work injury. 

Radiography 

A chest X-ray obtained incidentally revealed large lucencies in the right lung apex and mid-lung, consistent with emphysematous bullae. He was referred to a pulmonary clinic, where computed tomography (CT) imaging confirmed bilateral emphysematous bullae, worse in the right lung versus the left lung, secondary to COPD disease. 

Pulmonary Function Tests 

Approximately three years ago, he underwent pulmonary function testing, and spirometry showed that there was an obstructive ventilatory defect. Mid-expiratory flow was 1.35 L/second, 49% of predicted. Forced vital capacity (FVC) was 3.91, 103% of predicted, and forced expiratory volume in one second (FEV_1_) was 2.58 L, 87% of predicted. The FEV_1_/FVC ratio was obstructive at 66%. There was no improvement with the inhaled bronchodilator. Lung volumes by a body plethysmograph showed a normal total lung capacity of 5.83 L, 85% of predicted. There was a severe reduction in diffusion at 14.0, 50% of predicted. This was the only time he underwent pulmonary function testing. These results are detailed in Table 1 and demonstrate an obstructive pattern with reduced mid-expiratory flow and diffusion capacity, consistent with the patient’s known COPD.

**Table 1:** Pulmonary function test results demonstrating obstructive pattern with reduced diffusion capacity.

Pulmonary function test parameter	Result	Normal range	% Predicted	Units
Forced vital capacity (FVC)	3.91	3.0 - 5.0	103%	Liters (L)
Forced expiratory volume in 1 second (FEV₁)	2.58	2.4 - 4.0	87%	Liters (L)
FEV₁/FVC ratio	66	≥ 70	-	Percent (%)
Forced mid-expiratory flow (FEF25-75%)	1.35	2.3 - 3.5	49%	Liters/second (L/s)
Total lung capacity (TLC)	5.83	5.5 - 7.5	85%	Liters (L)
Diffusing capacity of the lung for carbon monoxide (DLCO)	14	22.0 - 32.0	50%	mL/min/mmHg

Over the last four years, he had used inhaled budesonide, glycopyrrolate, and formoterol fumarate, budesonide and formoterol fumarate dihydrate, and tiotropium bromide at different times for COPD control, although his adherence was inconsistent due to limited pharmacy access. Despite this, he required no hospitalizations for COPD exacerbations and was clinically stable. Based on his comorbidities and pulmonary status, he was classified as American Society of Anesthesiologists (ASA) physical status class III. 

Imaging

**Figure 1:**
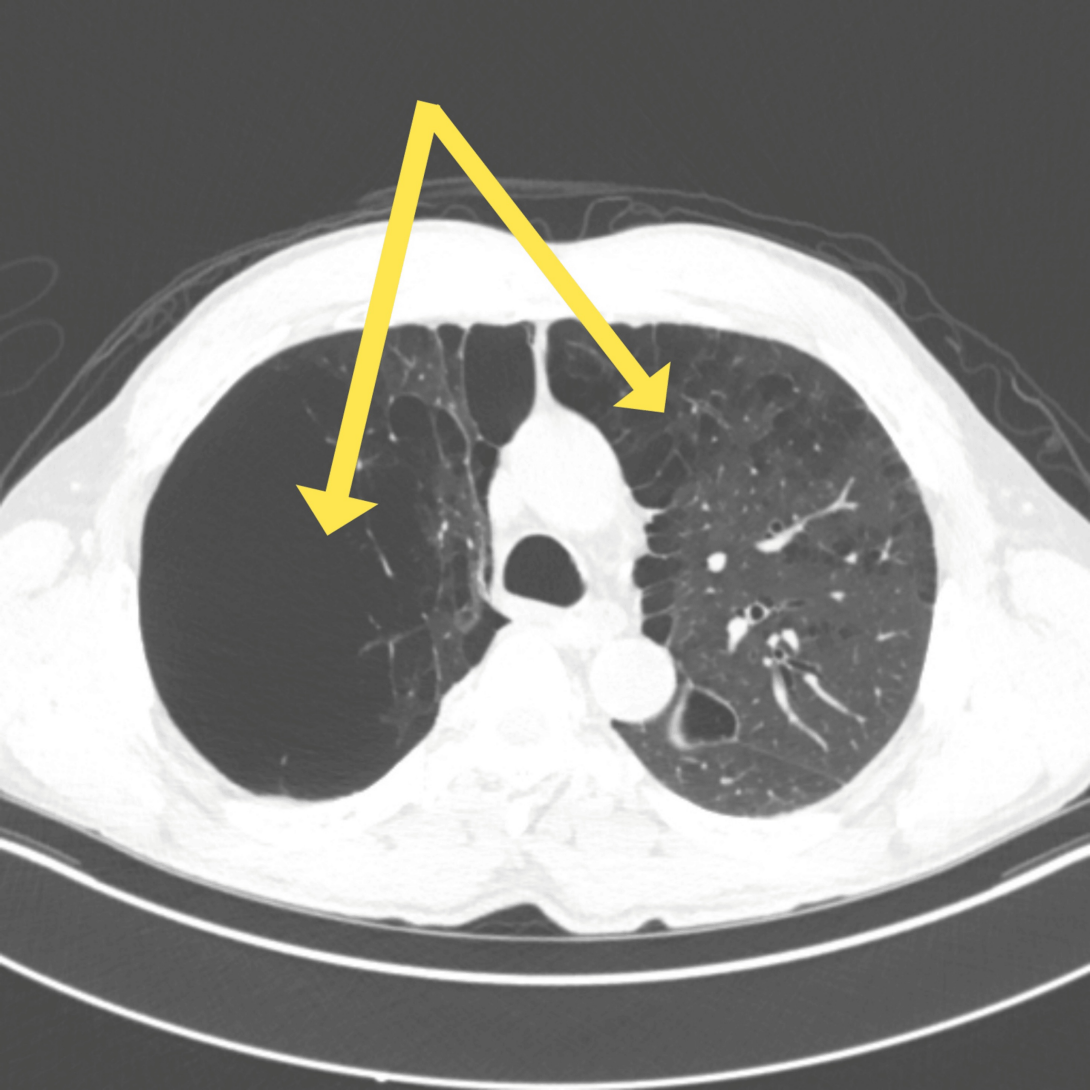
Preoperative axial chest computed tomography (CT) scan showing extensive bullous emphysema. The yellow arrows indicate giant bilateral bullae occupying the majority of the right upper and middle lobes.

**Figure 2:**
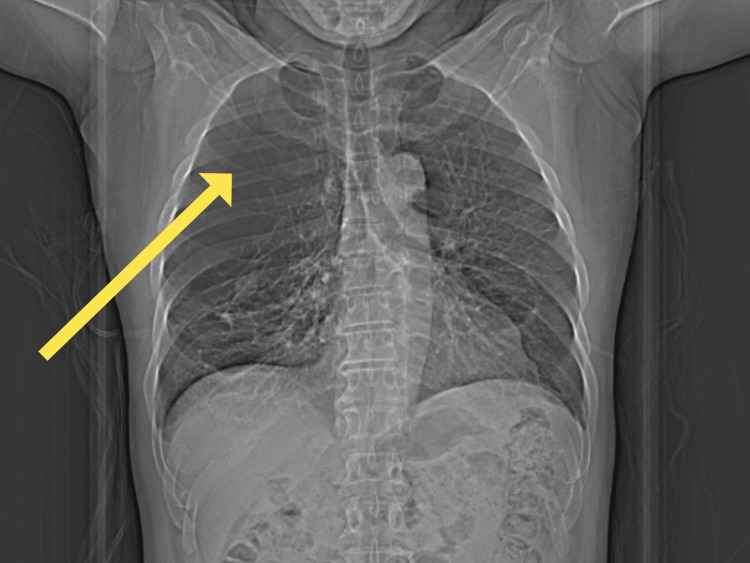
Preoperative chest X-ray demonstrating severe bullous emphysema. The yellow arrow indicates a large lucency in the right upper lung zone consistent with a giant bulla.

A preoperative CT of the chest revealed severe lung disease, extensive bilateral bullous emphysema, with nearly complete replacement of the right upper and middle lobes of lung parenchyma by giant bullae in addition to substantial bullous changes in the left lung, particularly in the apical segments, which were stable from his previous CT scans from pulmonology. These characteristic radiographic findings of severe bullous emphysema are depicted in Figures 1-2.

These findings raised concerns about the plan to do robotic laparoscopic hernia repair. The increased intrathoracic pressure and ventilation requirements due to positioning and CO_2_ insufflation for laparoscopy increased the risk of bullae rupture and barotrauma, including tension pneumothorax. The robotic nature of the surgery also created a physical barrier to intervention, as the docked robot would prevent anesthesia providers from promptly accessing the chest for decompression of a pneumothorax. 

After multidisciplinary consultation between the patient and the anesthesiology and surgery teams, the robotic approach was deemed unsafe. The decision was made to proceed with an open inguinal hernia repair under spinal anesthesia. 

Intraoperative course 

The patient was brought to the operating room after he received 2 mg of IV midazolam for anxiolysis through an 18-g peripheral IV in the left hand placed in the preoperative suite. A single-shot spinal dose of 1.7 mL of 0.75% bupivacaine was administered at the L3-L4 interspace using a 24-g spinal needle while the patient was in a seated position. Adequate sensory block and level was achieved. A 2 g dose of IV cefazolin was given for antibiotic prophylaxis, and pain control was supplemented with 1,000 mg of IV acetaminophen. 

A propofol infusion was given throughout the procedure for sedation at 75 mcg/kg/min to 50 mcg/kg/min with a total of 780.57 mg administered during the case. A 4 L/min of supplemental oxygen was provided via a nasal cannula. The patient remained hemodynamically stable with an uneventful intraoperative course. 

Postoperative course 

The patient was monitored postoperatively in the postanesthesia care unit (PACU) and remained stable. He was discharged and sent to an inpatient bed for observation. No postoperative pulmonary monitoring measures, such as repeat chest X-ray, were conducted. He was discharged to an outpatient adult drug and mental health facility on postoperative day two in good condition with no need for supplemental oxygen or further intervention. He was evaluated in the postoperative clinic three weeks afterward. He reported no complaints, and the hernia repair was healing as expected. 

## Discussion

Patients with emphysematous bullae found during preoperative assessment present unique challenges for the perioperative team. These bullae are large and thin-walled and are often susceptible to rupture under positive pressure ventilation. Previous literature suggests that these air spaces can be ruptured by different possible mechanisms based on the anatomy of the bulla and if it communicates with the bronchus. 

Bullae that are minimally communicative with the bronchus could rupture if nitrous oxide is used, causing expansion and rupture. If there is communication between the bulla and the bronchus, positive pressure ventilation with a high enough tidal volume may enter the bulla, or air may be progressively trapped inside the bulla with each respiration through a ball-valve effect until it eventually ruptures. Additionally, forceful exhalation or “bucking” during endotracheal tube manipulation could cause rupture of the unevenly thick walls of a bulla [1,7]. These possible mechanisms of rupture and subsequent barotrauma put significant responsibility on the anesthesiology and perioperative team to anticipate risk and optimize certain aspects of surgery and anesthesia strategy to mitigate these risks. 

In mechanically ventilated ICU populations, the overall incidence of barotrauma ranges from 6% to 13% in adults, with higher rates observed in patients with underlying lung pathology such as acute respiratory distress syndrome (ARDS) or COPD, which often includes emphysematous changes and bullae. For example, one prospective study found a barotrauma incidence of 6.5% in a general ICU population, decreasing to 0.98% with lung-protective ventilation strategies. In ARDS, the incidence can be as high as 13%. Patients with radiographic hyperinflation, often a surrogate for emphysematous changes, are at increased risk [8,9]. However, the literature does not provide a specific incidence for barotrauma exclusively attributable to rupture of emphysematous bullae. The risk is recognized to be higher in patients with preexisting bullous lung disease, but published data aggregate these cases with other forms of barotrauma. Therefore, the best available estimate is that the incidence of barotrauma in mechanically ventilated patients with underlying lung disease may be in the range of 6-13%, but the precise proportion due to bullae rupture alone is not delineated in the current literature [8,9]. 

Preoperative assessment

Imaging 

Preoperatively, reviewing prior chest imaging such as X-rays and CT scans can be critical in identifying bullae for patients with significant risk factors. In this case, our patient had documented severe emphysematous changes from COPD, and additional risk factors such as a long history of tobacco and marijuana usage. This preoperative risk assessment imaging was key in avoiding possible complications. 

Pulmonary Function Tests 

It is important to note that severe bullous changes can be present even in patients with normal or stable pulmonary function test results, and severe changes have been noted incidentally in patients with no previous diagnostic imaging or pulmonary function testing [10]. 

Risk Scoring Tools 

Additionally, risk assessment tools such as the Assess Respiratory Risk in Surgical Patients in Catalonia (ARISCAT) may underestimate the risk of all pulmonary complications in these patients [11,12]. As such, although it is a valuable tool, it is only one part of a comprehensive risk assessment for these patients, and risk assessment for intraoperative complications may require including an individualized approach that incorporates imaging and anatomy. 

A possible limitation in our case is the absence of updated pulmonary function testing and recent imaging immediately prior to surgery, which may have offered additional detail to risk stratification and preoperative planning.

Ventilation-Perfusion (V/Q) Scan

When feasible, a V/Q scan can further inform perioperative risk by determining the anatomy of the bulla and whether it communicates with the bronchus. In one case report, a prior V/Q scan before bilateral bullectomy identified the patient’s bulla as non-communicating, and the perioperative team considered the risk of distension of the bulla to be reduced. The team used sequential one-lung ventilation with gentle pressure below 15 mmHg, and the procedure was completed with no complications [7]. Strategies such as this may be able to further stratify patients based on risks specific to their specific bulla anatomy. Currently, V/Q scans are recommended as preoperative work-up for these patients; however, their utility for preoperative risk assessment remains relatively unexplored in the literature [13]. 

Multidisciplinary Consultation for Possible Optimization and Intervention 

There may be value in early referral to interventional pulmonology or thoracic surgery for evaluation for definitive therapies for these patients. In certain cases, prophylactic intervention prior to elective surgery may reduce intraoperative risk. Procedures such as prophylactic bullectomy, which have been shown to reduce rates of pneumothorax immediately postoperatively after specific surgeries, may be discussed before surgery [14]. Other interventions classically used for chronic symptomatic control of emphysema and COPD, such as lung volume reduction surgery or endobronchial valve placement, may also confer some benefit in reducing intraoperative risk by removing the bullous lobes of affected lungs or by deflating giant bullae using Zephyr valves [15]. Although there is evidence that these options are sometimes considered before surgery for risk mitigation, they have not been extensively investigated for their role in preoperative optimization for ventilation during surgery [16]. 

Intraoperative management 

Surgical Approach 

Key intraoperative aspects of the surgery for these high-risk patients should be optimized when possible. In our case, the original recommendation of robotic laparoscopic surgery would have limited provider access to the chest. Hence, this risk was conveyed to the surgical team, and the surgical modality was quickly changed to open hernia repair. Cases where bullae have ruptured in robotic surgery have been reported in the literature, and it is possible that robotic platforms could increase the time required for intervention for pneumothorax [6]. Other risk mitigation strategies include having instruments for chest tubes immediately available and heightened intraoperative vigilance for signs of pneumothorax [16,17]. In one case, two providers were assigned to auscultate the lungs of a high-risk patient to immediately identify early signs of pneumothorax if it had occurred [9]. 

Anesthesia Choices 

The choice of anesthetic strategy is also a variable that can be tailored to the patient. In our case, general anesthesia and positive pressure ventilation were able to be completely avoided using monitored anesthesia care under propofol infusion and the use of a spinal block. Several reported cases of similar strategies have been described in the literature using regional nerve blocks as the primary anesthetic approach [18,19]. Additionally, another utilization of regional anesthesia is described in one report following spine surgery, where a transtracheal block was administered at the end of the case to minimize coughing and bucking during emergence to reduce the risk of bulla rupture [20]. These examples demonstrate the utility of regional anesthesia as both the primary anesthetic strategy and as an adjunct for this patient population. 

Ventilatory Strategies 

Several lung protective ventilatory strategies that can be employed to minimize the risk of bullae rupture and pneumothorax are reported in previous literature. Common techniques used include lung protective strategies, taking care to maintain lower tidal volumes, and ensuring peak inspiratory pressures remain below 20 cm H_2_O or 15 cm H_2_O to minimize the positive pressure and volume bullae are exposed to [10,16,17]. However, in patients with high lung compliance, even low airway pressures may still transmit significant force to these structures, possibly maintaining a risk of rupture despite conservative settings. Another strategy is to allow for spontaneous ventilation after intubation when possible [18,20]. In cases where bullae are asymmetrically distributed bilaterally or bullectomy is planned, one-lung ventilation with low pressure and volume has also been used with success [9]. Regarding non-ventilation-related concerns, a common consideration is avoiding nitrous oxide during surgery due to the risk of diffusion into and subsequent expansion of bullae [10,16,20]. While chest tube placement is often considered a readily available and effective treatment for pneumothorax, reliance on it as a fallback may not be effective in treating pneumothorax and subsequent lung expansion. In patients with large emphysematous bullae, such as this case, the communicating fistula between the emphysematous bullae and the bronchus is large, and in the event of bullae rupture, the volume of air escaping to the pleural cavity may overwhelm the drainage capacity of the chest tube, causing worsening of barotrauma and hypoxia. 

Finally, considerations should be made for smooth intubation and extubation to prevent increased airway pressure from forceful exhalation or bucking. Approaches for this purpose include administering topical anesthesia before extubation in addition to airway anesthesia, such as the transtracheal local anesthesia administration before airway management [10,20]. 

## Conclusions

Emphysematous bullae in patients undergoing anesthesia present unique challenges and sometimes life-threatening risks that require vigilant preoperative risk assessment and communication between perioperative teams to determine the safest surgical and anesthesia approach to patient care. Preoperative risk assessment for these patients may need to go beyond standard tools and should include individualized evaluation and imaging. The importance of preoperative consultation with pulmonology cannot be overstated, as they may offer preoperative risk reduction interventions, including bullectomy, lung volume reduction modalities such as endobronchial valves, or surgery. Regional anesthesia is an anesthetic option that can be utilized to avoid intermittent positive pressure ventilation (IPPV) and reduce the risk of barotrauma from potential bullae rupture. In high-risk emphysematous patients, regional anesthesia should be considered early, and not only as a fallback after failed induction attempts. It should be noted that in patients requiring general anesthesia with IPPV, relying on chest tubes as a fallback intervention in case of pneumothorax may offer a false sense of security. Especially in our case with a large communication between the bullae and the bronchus, continuous airflow into the ruptured bulla may overwhelm the ability of a chest tube to adequately decompress the pleural space. Instead, it is important to optimize the intraoperative ventilation strategy. 

While this case highlights several strategies used in high-risk patients, the choice of specific interventions should be guided by the individual’s anatomy, disease severity, and procedural context. This case adds to the growing body of literature describing unique perioperative challenges and choices to avoid complications in this high-risk surgical patient population. 
